# Advancements in Nanotechnology for Spinal Surgery: Innovations in Spinal Fixation Devices for Enhanced Biomechanical Performance and Osteointegration

**DOI:** 10.3390/nano15141073

**Published:** 2025-07-10

**Authors:** Bogdan Costăchescu, Elena-Theodora Moldoveanu, Adelina-Gabriela Niculescu, Alexandru Mihai Grumezescu, Daniel Mihai Teleanu

**Affiliations:** 1 Department of Neurosurgery, “Gr. T. Popa” University of Medicine and Pharmacy, 700115 Iasi, Romania; bogdan.costachescu@umfiasi.ro; 2 ”Prof. Dr. N. Oblu” Emergency Clinical Hospital, 700309 Iasi, Romania; 3 Department of Science and Engineering of Oxide Materials and Nanomaterials, Politehnica University of Bucharest, 011061 Bucharest, Romania; elena.moldoveanu99@upb.ro (E.-T.M.); adelina.niculescu@upb.ro (A.-G.N.); 4 Research Institute of the University of Bucharest—ICUB, University of Bucharest, 050657 Bucharest, Romania; 5 Department of Neuroscience, “Carol Davila” University of Medicine and Pharmacy, 050474 Bucharest, Romania; daniel.teleanu@umfcd.ro

**Keywords:** spinal implants, rods, screws, spinal fixation devices, nanotechnology, osteointegration

## Abstract

Spinal injuries have a major impact on patients’ quality of life due to the implacable consequences they bring, such as reduced mobility and loss of flexibility, in most cases requiring surgery to restore spinal stability and functionality. In this respect, spinal fixation devices represent an important strategy to stabilize the spine after severe injuries or degenerative conditions, providing structural support and preserving spinal function. However, at the moment, the materials used to manufacture spinal implants present numerous disadvantages (e.g., Young’s modulus larger than cortical bone, which can produce bone resorption and implant enlargement) that can lead to implant failure. In this context, nanotechnology can offer promising solutions, bringing improved properties (e.g., biocompatibility, osseointegration, and increased mechanical performance) that increase the potential for obtaining devices customized to patients’ needs. Thus, the present work aims to present an overview of the types of nanocoating surface modification, the impact of rough and porous implant surfaces, and the integration of bioactive nanoparticles that reduce the risk of infection and implant rejection. In addition, incorporating 3D printing technology and the use of biodegradable materials into the discussion provides a valuable perspective for future studies in this field. Although the emerging results are encouraging, further studies to assess the long-term safety of implant coatings are needed.

## 1. Introduction

Spine disorders (SD), such as spinal injuries, degenerative diseases, and spinal deformities, represent major musculoskeletal conditions caused by various factors (e.g., daily habits, posture conditions, comorbidities, and traumas) that can lead to disability [1,2,3]. They are characterized by severe pain and have a tremendous socioeconomic impact, reducing patients’ quality of life [1,2]. Consequently, managing SD generally involves physical examination and imaging techniques, non-operative treatments that are sufficient [4]. However, in other cases, clinical practice implies surgical approaches to maintain normal or near-normal motion [5].

Preserving the mechanical stability of the spine has become a priority for achieving successful surgical outcomes, with researchers producing more and more diverse and reliable implants that possess properties similar to bone (e.g., Young’s modulus, fatigue strength), while ensuring imaging compatibility and biological inertness [6,7]. Commonly used spinal devices includes multiple types of implants for fusion and fixation of the spine, such as screws, spine fixation plates, spinal fixation rods, clamps, hooks, cages, and cervical artificial disk replacements [8,9]. Implantable devices for spinal surgeries require constant improvements to address the following conditions: (1) to achieve adequate mechanical properties that maintain spinal stability, (2) to develop materials with increased market potential and high biocompatibility, and (3) for the implants to be biostable [7].

These implants (Figure 1) can be designed with metals, polymers, ceramics, and composites since these materials can span a range of properties different enough to meet the desired needs.

For this purpose, nanotechnology can be used to provide and develop new materials with enhanced properties [4,5,14]. In this regard, nanotechnology is currently involved in providing a growing area of research [7,8,9,10,11,12,13,15]. For spinal implants, nanotechnology can be employed to modify various features, such as hydrophilicity and overall roughness of the implant’s surface, toward promoting osseointegration [16,17]. Henceforth, with the development of modern spinal implants that possess osteoinductive and osteoconductive properties, the ability to create novel bone grafts that achieve solid fusion has advanced significantly [18]. Moreover, nanotechnology can be applied to enhance surgical outcomes, promote adequate and improved bone healing, and reduce complications associated with orthopedic interventions by extending the life of implants, improving their biocompatibility, treating osteoporotic vertebral fractures, preventing infections, treating orthopedic oncology, and using stem cells for regenerative medicine [19,20,21,22].

Figure 2 illustrates the evolution of materials used in spinal implants, highlighting the introduction or development of new materials aimed at enhancing their biomechanical performance and biocompatibility. Moreover, this figure highlights the transition from rigid materials to new, innovative materials that can be personalized for patients’ needs. In this respect, materials used in spinal implants, such as metals (e.g., titanium and titanium alloys, stainless steel, and cobalt-chromium), ceramics (e.g., silicon nitride), and polymers (e.g., polyether ether ketone), need to provide a balance between physical properties and biocompatibility. Metallic materials are preferred in the fabrication of implantable materials due to their superior mechanical properties; however, their biocompatibility needs improvement. In this regard, the primary goal is to prevent harmful reactions post-implantation, such as stress shielding or poor interaction between the implant surface and tissues [23,24]. Ceramic ones exhibit higher biocompatibility and mechanical strength, reducing adverse reactions and inflammation, and possess bioactive potential. They are preferred for designing permanent implants. In contrast, polymers have low immunogenic properties, high biocompatibility, but poor mechanical properties [24,25]. The surface physicochemical properties of implants have significantly improved their mechanical properties and biocompatibility with bone tissue, two critical criteria for implant materials, thereby decreasing the risk of implant failure [26,27]. Thus, using materials with high surface area and improved physicochemical properties, implant rigidity can enhance biological interactions, such as adhesion, propagation, bone-related protein synthesis, and mineral accumulation [28].

This narrative review aims to explore advances in nanotechnology and establish a current framework for developing novel materials for spinal implants. In this respect, the paper begins by outlining the requirements for the biomechanical and biological properties of implants, and then discusses innovations in the field, including the use of 3D printing and biodegradable materials. Current challenges and future perspectives also provide a comprehensive overview of the research directions and clinical applicability of the identified emerging solutions. In this respect, recent English-language papers were selected and analyzed in this review. The information was gathered from scientific databases such as Google Scholar, PubMed, MDPI, Science Direct, Scopus, SpringerLink, and ResearchGate, using a variety of combinations between the following keywords: “spinal implants”, “spinal surgery”, “nanotechnology”, “nanomaterials”, “nanostructured devices”, “nanocoatings”, “spinal screws”, “pedicle screws”, and “implant osseointegration”.

## 2. Biomechanical and Biological Requirements for Spinal Implants

Although the spine represents a robust structure able to support the entire body’s weight, it has considerable flexibility, enabling the necessary mobility for motion and daily activities [32]. Flexibility varies depending on the spine region and is influenced by the anatomy of the vertebrae, intervertebral disks, and the mobility requirements of different body parts. For example, the cervical region requires high mobility to help the head move and allows a wider field of vision. In contrast, the thoracic region is more rigid than other regions. In contrast, the lumbar region is relatively flexible in flexion-extension movements, especially in the lower segments [33]. The differences between the specific regions of each spine segment are also reflected in the distribution of the biomechanical loads they bear during physical activity. Table 1 shows the physiological loads exerted on the spine presented in the literature.

Thus, the loads produced by compression and bending vary depending on the spinal segment and activity. At the same time, activities such as bending forward or getting up from the chair produce the most significant stress on the spine. Thus, some spinal damage can considerably reduce its mobility and increase its stiffness. In this regard, implants should mimic the spine’s biomechanical properties [33].

Thus, tensile strength, fatigue, elastic modulus, and elongation of biomedical materials are essential factors for load-bearing applications in hard-tissue implants [38]. Implants’ load-bearing capacity can be influenced by factors such as their strength [38,39]. According to Table 1, the forces exerted on the spine during physical activities (e.g., compression, flexion, lifting) considerably influence the choice of materials (Table 2) to fabricate spinal implants. Thus, for the cervical region, the materials should have a low modulus of elasticity with adequate flexibility to mimic physiological mobility. In contrast, for the thoracic region, the flexibility is lower, but the compression is higher. For the lumbar region, materials with high resistance to compression and fatigue are required, which, at the same time, have efficient osseointegration, as this is the most stressed region. In this respect, metallic materials are generally used in spine implant manufacturing because of their high mechanical strength, ductility, formability, corrosion resistance, and toughness, which are more suitable in orthopedic applications than ceramics or polymeric materials [39,40,41]. Yet, these materials have disadvantages such as a higher Young’s modulus than cortical bone, producing stress shielding, bone resorption, and implant loosening [42]. However, even if metallic materials (e.g., stainless steel, Co-Cr alloys, tantalum) are strong, they are too stiff. In contrast, more flexible materials like polymers have low mechanical strength, and ceramics are too brittle. Thus, composite materials represent an advanced, more convenient strategy [7,43,44,45].

However, corrosion, fatigue, and wear can also influence implant service life and are considered major drawbacks that produce implant loosening, stress shielding, and implant failure [38,39,46]. When corrosion occurs, it increases the frictional wear that can release undesirable metallic ions and induce cytotoxic reactions in patients [38,47]. Corrosion residues are detected by the human body as pathogens, thus activating an immune response that disrupts local homeostasis and causes acute inflammation, leading to the formation of irreversible fibrous capsules around the implants, affecting the integration of the device and favoring implant failure [47,48].

Moreover, the development of spinal implants requires not only the selection of materials but also the development of manufacturing techniques, which can enhance both the mechanical and biological properties. In this regard, two major methods (Figure 3) are used: subtractive manufacturing and additive manufacturing processes [49,50,51].

**Table 2:** Characteristics and applications of spinal implant materials.

Category	Class	Material	Applications	Properties	Advantages	Disadvantages	**Refs.**
Conventional materials	Metals	Titanium and its alloys	Screws, rods, cages	Elastic modulus: 110 GPa Yield strength: 789–1013 MPa Fatigue limit: 500–600 MPa	High strength MRI-compatible Corrosion-resistant Promote osseointegration	Too stiff -> stress shielding Poor multilevel fusion Produces imaging artifacts	[7,38,39,44,52,53,54]
		Stainless steel and its alloys	Rods, screws	Elastic modulus: 200 GPa Yield strength: 690 MPa Fatigue limit 350–500 MPa	High strength Low-cost fabrication	Poor biocompatibility Low corrosion resistance Risk of cracking	[7,39,54,55,56,57,58]
		CoCr and its alloys	Rods, screws	Elastic modulus: 200–300 GPa Yield strength: 800–950 MPa Fatigue limit: >600 MPa	High hardness Wear resistance, fatigue strength Relatively expensive High ductility	Stiffness Allergic potential Produces imaging artifacts	[7,39,40,54,55,57,58,59]
		Tantalum	Screws, rods, cages	Elastic modulus: 3 GPa Yield strength: 789–1013 MPa Fatigue limit: 500–600 MPa	Biocompatible Porous Promotes fusion	Limited availability High-cost production High melting point High chances of infections post-implantation	[7,54,60,61,62,63]
	Polymers	Polyetheretherketone (PEEK)	Cages, rods	Elastic modulus: 3.6 GPa Yield strength: 165 MPa Fatigue limit: 99.4–107.4 MPa	High stability High strength Good wear resistance and fatigue properties Non-toxic Elastic modulus similar to cortical bone tissue Reduce the extent of stress shielding	Poor osteointegration Risk of loosening/migration	[7,64,65,66,67,68]
		Polylactic acid (PLA)	Cages	Elastic modulus: 3500 GPa Yield strength: 60 MPa Fatigue limit: 13.7 MPa	Biocompatible and bioresorbable Does not require surgery to remove the implant	Low bioactivity Low strength	[69,70,71,72,73]
		Poly (vinyl alcohol) (PVA)	Replacement in the intervertebral disk herniation	Elastic modulus: 0.0012–0.85 MPa Tensile strength: 1.73 GPa	Flexible Biocompatible	Low mechanical stability	[74,75]
Innovative Materials	Ceramics	Bioglass	Cages	Elastic modulus: 13 ± 2 GPa Yield strength: 253.34 ± 9.31 MPa Fatigue limit: 30 MPa	Promotes bone integration Good radiological outcomes	Less effective than autograft Limited long-term data	[7,76,77,78,79]
		Silicon Nitride	Cages	Elastic modulus: 236 ± 10 GPa Yield strength: 65.3–127 GPa Flexural strength: 0.750 GPa	Osteoinductive Non-toxic Has antimicrobial properties High strenth	Large-scale clinical trials are limited High production costs	[7,80,81,82,83,84]
		Apatite Wollastonite	Cages	Elastic modulus: 32 GPa Maximum compressive strength: 121 MPa	Bioactive Biocompatible Biodegradable Induces osseointegration Reduce stress on the implant	Large-scale studies are limited	[7,85,86]
	Metals	Nitinol	Cages, rods, screws, supporter bands	Elastic modulus: 48 GPa Yield strength: 1050 MPa Tensile strength: 1521 MPa	High mechanical resistance Corrosion resistante Low corrosion rate Induce osseointegration Osteoinductive	Nickel toxicity concern Large-scale clinical trials are limited	[7,87,88,89,90]
	Composite	Carbon-fiber-reinforced (CFR)-PEEK	Pedicle screw, cages, vertebral body replacements, rods	Elastic modulus: 18 GPa Compressive strength: 301.00 ± 1.27 MPa Flexural strength: 728.25 ± 22.5 MPa	Customizable stiffness Artifact-free imaging Enhance artifact-free imaging to evaluate therapeutic success Their mechanical properties depend on the carbon fibers’ amount resulting in tunable mechanical properties Roods can provide effective primary stability Screws have higher pullout strength Carbon-fiber cage has shown safety and durability	Torsional stiffness and yield torque are lower Increased chance of bacterial adhesion High risk of screw loosening	[91,92,93,94,95]

## 3. Nanotechnology in Spinal Implant Materials: Innovations and Applications

The main disadvantage of medical implants is the risk of failure or dysfunction, attributed to different factors (e.g., infections, poor mechanical properties, corrosion, bone density, and tissue rejection), which can lead to complications and adverse health outcomes [96,97]. Nanomaterials can improve properties such as bone biogenesis, cell adhesion, and accumulation of calcium minerals by modifying the physicochemical characteristics of the materials used in implant manufacturing, such as smoothness, increased stiffness, and increased surface area [98]. Thus, the chemical composition, hydrophilicity, and overall roughness achieved with nanotechnology play a key role in bone fusion. Recent research has focused on surface modifications, using chemical, physical, and biological methods (Figure 4) that offer improved immunomodulation without altering the bulk material, providing enhanced biocompatibility [51,99,100].

Given the limitations of implants, nanotechnology continues to develop and optimize coatings that will address these challenges. Thus, nanocoatings are obtained by deposition of an ultrathin layer (typically <100 nm) on implant surfaces, having enhanced properties or imparting new functionalities. Nanocoatings typically involve metallic nanoparticles (e.g., silver, copper, gold, zinc) and non-metallic nanoparticles (e.g., graphene, carbon nanotubes, polymeric materials). Also, combining nanoparticles with enhanced properties creates a composite that produces nanocoatings with bioactive, osteogenic, antibacterial, and biodegradable properties, creating bioactive coatings [102,103,104,105]. In this respect, bioactive coatings (Figure 5) have begun to be developed to enhance the biological performance of implants [100,106,107,108]. However, using nanocoatings in medical implants still represents an early stage in the field, although there has been significant progress in recent years [96].

Nanoscale surface structuring enables cellular interaction, promoting bone formation by modification of the surface and bulk characteristics of implants [6,109]. This approach has been demonstrated to maximize osseointegration and long-term stability by introducing intrinsic porosity and surface roughness and by observing its effects on spinal interbody mechanics, vascularization, osteoblast attachment, and ingrowth potential [109]. It was observed that both micro (3–30 µm) and nanoscale (<100 nm) roughness have been found to facilitate osteoblast attachment and differentiation, while reducing micromotions between the implant surface and bone. In addition, it was found that roughness leads to the stimulation of osteogenic (BMPs, TGF-β), antiresorptive, and angiogenic (VEGF, FGF-2, angiopoietin-1) factors [110,111,112]. Additionally, implants with porosities ranging from 30 to 90% are associated with superior osseointegration and vascularized bone formation. Pore sizes are important, and those between 100 and 600 µm have been observed to be optimal. However, pore interconnectivity is also essential for nutrient exchange and cell penetration [110,113].

**Figure 6:**
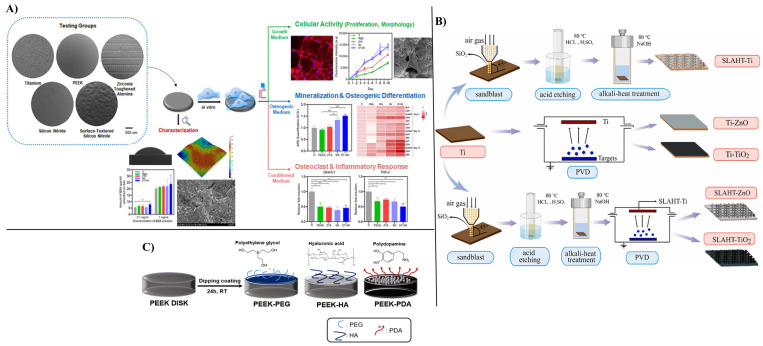
Comparative study of biomaterials for spinal implants adapted based on open access sources: (**A**) Schematic representation of implant surface modification (Ti, PEEK, silicon nitride, surface-textured silicon nitride) realized by Lee et al. [124]. (**B**) Surface modification strategy for polished pure titanium, realized by Ma et al. [125]. A combination of sandblasting, acid etching, and hydrothermal treatment (SLAHT) was used to create textures. Separately, sputtering was used to deposit layers of ZnO and TiO_2_ on smooth, untextured Ti implants. Finally, the two methods (SLAHT and sputtering) were combined to apply layers of ZnO and TiO_2_ to Ti implants with complex textured surfaces. (**C**) Fabrication strategy, introduced by Park et al. [126] of PEEK cages through the deep-coating method using polyethylene glycol/hyaluronic acid/polydopamine for surface modification. Images reprinted from the mentioned open access sources.

**Figure 1:**
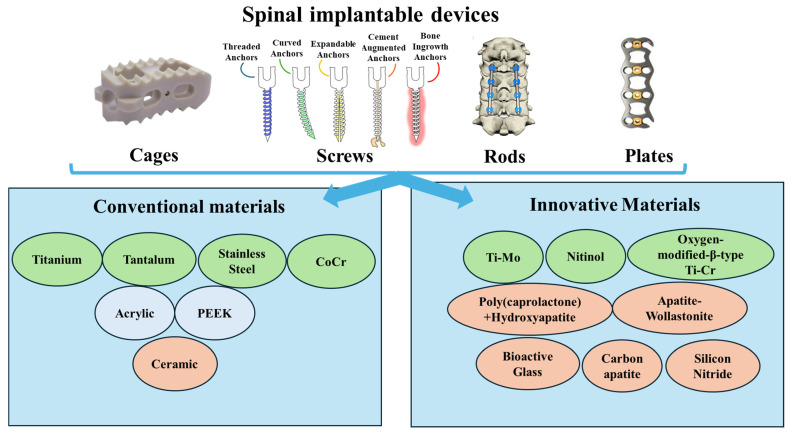
Spinal implants: device types and material evolution. Created based on the information from [7,10,11,12,13]. Green—metallic materials; orange—ceramic materials; and light blue—polymers.

**Figure 2:**
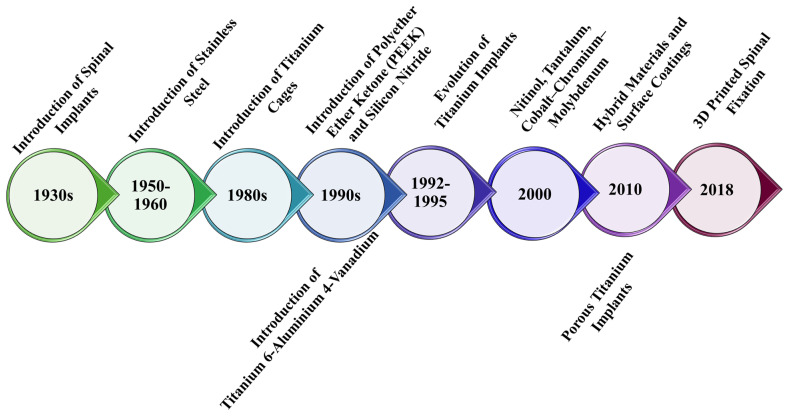
Timeline of materials used in spinal fixation devices manufacturing. Created based on the information from [29,30,31].

**Figure 3:**
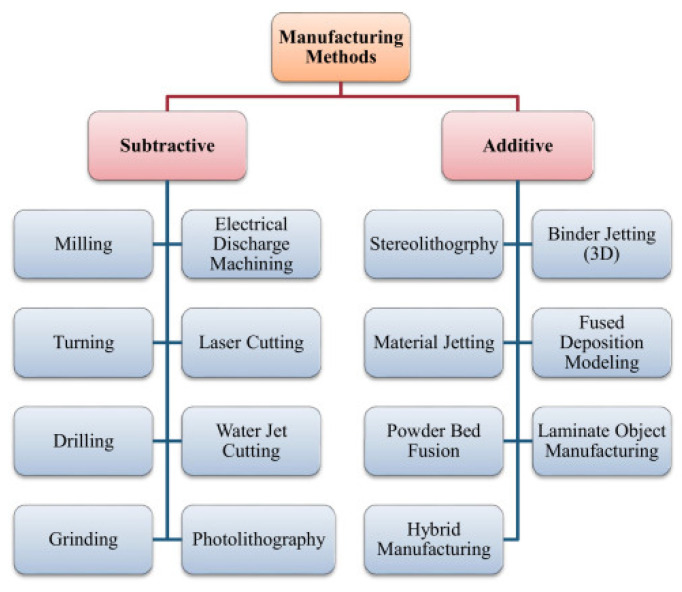
Schematic representation of the implant fabrication processes. Reprinted from an open access source [49].

**Figure 4:**
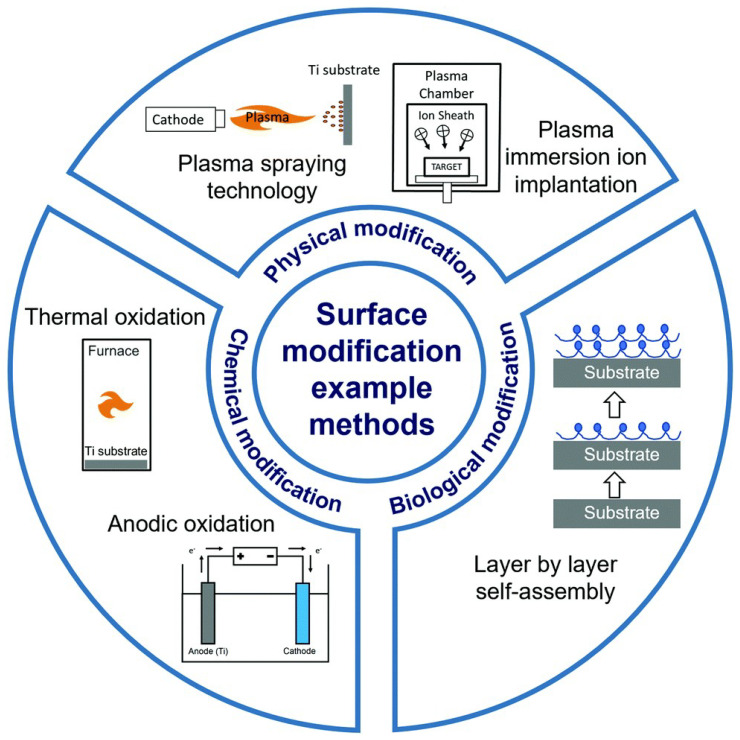
Surface modification techniques for implants. Reprinted from an open-access source [101].

**Figure 5:**
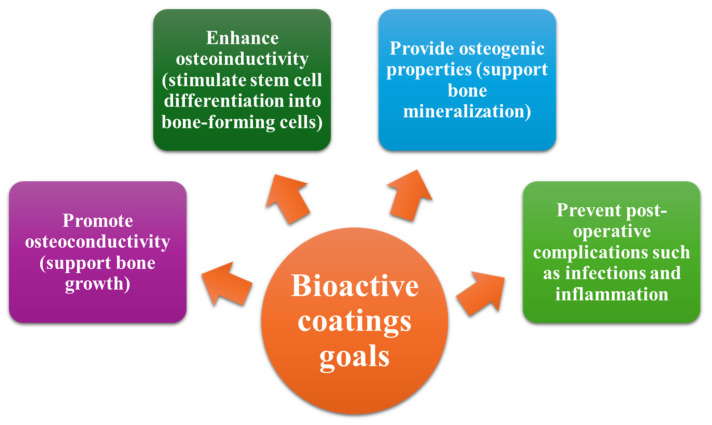
The primary goals of bioactive coatings. Created based on the information from [106].

Nanomaterial-based surface modifications play a key role in enhancing the biocompatibility of spinal implants by improving cellular interactions, protein adsorption, and tissue integration. In this regard, Table 3 compares and summarizes information about various materials used for spine implant manufacturing and surface modification strategies. Moreover, selected strategies are illustrated in Figure 6 to offer an at-a-glance perspective. Thus, developing a coating that protects implant surfaces in the harsh environment of the human body is needed [114,115]. For example, additive manufacturing techniques are leading to layer-by-layer construction using ceramics (e.g., hydroxyapatite (HAp), metals (silver (Ag), gold (Au)), and metallic oxides (e.g., TiO_2_, ZrO_2_)) and polymers (e.g., poly L-lactic acid) to solve these drawbacks [6,51]. These coatings enhance the biocompatibility of implants by mimicking the natural nanostructure of bone, reducing inflammatory responses, and promoting implant osseointegration [116,117]. In titanium and titanium alloy-based implants, titanium dioxide layers play a crucial role in enhancing their biocompatibility, thereby promoting bone growth in spinal cages. Also, it has been demonstrated that surface modification, such as micro- and nano-texturing, improves osteoblast activity [118,119]. This analysis emphasizes the importance of the interaction of surface topography, functionalization, tissues, and cellular responses to obtain modern, safe, and efficient spine implants. For example, a study by Nathaniel Toop et al. [120] showed that the roughness created on titanium surfaces using surface acid-etching improved osteoblast differentiation and angiogenesis, having superior osseointegration compared with PEEK-based implants. In this case, titanium cages significantly promoted early fusion rates compared with PEEK cages. Bioglass coatings (e.g., calcium silicate ceramics, silicon nitride) are used to enhance the osseointegration of implants, promoting faster bone fusion [118].

Moreover, postoperative infections also represent a significant concern; therefore, studies have investigated the relationship between infection and implant failure rates. Thus, low-grade infections can be considered as a possible cause of implant loosening and failure in patients with spinal instrumentation. Early detection and treatment of these infections could improve surgical outcomes and reduce the need for revision surgeries [121,122,123].

**Table 3:** Modern surface modification strategies for implant materials: comparison of materials, methods, and biological responses.

Material Type	Implant Surface	Surface Modification	Surface Modification Methods	Biological Activity	Limitations	**Refs.**
Titanium	Titanium Alloy (Ti6Al4V) Disks	Roughness	Sandblast, large grit Acid-etching	Ti has one of the highest rough surfaces Ti showed a proinflammatory response	Inflammatory markers increased on Ti This surface has a low apatite formation	[124]
	Pure Titanium Disks	Hybrid coating Antimicrobial peptides GL13K+ silver nanoparticles (AgNPs)	Acid-etching and immersion	Antimicrobial efficacy against *S. gordonii*, *MRSA*, and *P. aeruginosa* No cytotoxic effect on hBMSCs cells In vivo tests showed reduced inflammation	Long-term stability, integration, and toxicity must still be evaluated in future work	[127]
	Titanium Substrate	Rough, porous surface Coating with ZnO and TiO_2_ nanoparticles	Sandblast, Acid-etching, and Hydrothermal Treatment (SLAHT)	Enhanced roughness Coating promoted cell viability in L929 cells No cytotoxic effects observed A high antimicrobial effect was provided	Long-term stability, integration, and toxicity must still be evaluated in future work	[125]
	Titanium Alloy Cages	Ag-HA coating	Not reported	Improved osseointegration Prevented infection No Ag-related complications	There were no control groups Short time of trial Small sample size Long-term, large-scale trials are needed	[128]
	Pure Titanium Pieces	Roughness Coating based on Ag-HA	Sandblast Thermal spray technique	Ag-HA provided enhanced osteoconductivity and improved bone contact -> improved spinal fusion No neurotoxic effect was noticed Ag-HA coating represents a potential biologically safe strategy	Short-term study (8 weeks) Silver accumulation in other organs still needs evaluation	[129]
	Titanium Implants	Coating based on HA substituted with silver (Ag^+^) and strontium (Sr^2+^)	CoBlast	Sr-HA promoted MG-63 cell metabolic activity, compared with the other coatings Ag-HA showed significant antimicrobial efficacy and inhibited biofilm formation	Ag-Sr-HA needs optimization Long-term stability, integration, and toxicity must still be evaluated in future work	[130]
PEEK	PEEK Disks	None	As Machined	Low inflammation	Poor cellular adhesion due to high hydrophobicity Showed low mineralization and osteogenic gene expression	[124]
	PEEK Substrate	Bioactive coating with strontium-modified Eucommia ulmoides polysaccharides (EUP-Sr) Porous structure	Not reported	Enhanced MC3T3-F1 proliferation, adhesion, RUNX2 and Col1-α1 expression, ostegenic and anti-inflammatory	Higher concentration of UP-Sr can produce cytotoxicity	[131]
	PEEK Disks	Nanocoating with osteogenic and antimicrobial properties Graphene oxide (GO) nanosheets, Polydopamine (PDA) nanofilm, and bone-forming peptide (BFP)	Immersion coating	Promoted osteoblast proliferation Promoted apatite formation High antimicrobial efficacy	Long-term stability, integration, and toxicity must still be evaluated in future work	[132]
	PEEK Disks and Intervertebral Cages	Coating based on PEG, HA, PDA	Immersion coating	All the coatings promoted osteoblast proliferation	Long-term stability, integration, and toxicity must still be evaluated in future work. No significant differences between mechanical performances	[126]
Silicon nitride	Silicon Nitride Disks	None	As-Fired	Silicon nitride presented the best apatite formation and promoted cell proliferation High protein adsorption Biomimetic aspect	Long-term stability, integration, and toxicity must still be evaluated in future work. No significant differences between mechanical performances	[124]
	Surface-Textured Silicon Nitride Disks	Roughness	Laser-patterned surface			
Stainless Steel	Stainless Steel Plates	Niosomes—nonionic vesicular nanocarriers Vancomycin-loaded niosomes	Layer-by-layer technique Dip-coating	Sustained antibiotic release (28 h) Reduced bacterial adhesion and colony formation No cytotoxic effect was observed on L929 cells	Long-term stability, integration, and toxicity must still be evaluated in future work	[133]

Additionally, Table 4 provides a comprehensive comparison of surface modification types used to enhance the performance of implantable materials. Thus, the table specifies the type of spine implant, the surface treatment, and biological and mechanical effects. Regarding screw loosening and pull-out effects, which represent the main failure of spinal implants, especially in patients with osteoporosis or under high loads, nanotechnology can enhance the performance and durability of implants by reducing friction, improving adhesion, and preventing material corrosion and wear [6,134]. Roughness increases resistance to the pull-out effect due to improved initial fixation, while porosity reduces stress shielding in implants [134,135].

## 4. Limitations, Future Perspectives, and Emerging Trends

Despite recent progress, extensive research is still required to optimize the design and materials used in spinal implants. Although nanotechnology can bring considerable progress in developing and improving spinal implants, it also brings challenges and risks. Firstly, spinal implants cannot facilitate the full recovery of structural integrity, biomechanical function, and kinematics, and are also limited by the material’s physicochemical properties. Thus, concerns regarding the production of wear products and their biological effects have recently started to attract attention, being responsible for late-onset spinal pain, hypersensitivity, cytotoxicity, inflammation, osteolysis, and pseudotumor formation [137]. However, inert materials are also associated with weak osteointegration, producing effects such as local systemic toxicity due to ion release and the development of a fibrous collagenous capsule around the implant [138]. Composites and surface modification used on the materials’ physicochemical properties of implant surfaces can be manipulated to match natural bone. However, even if the scope is to improve osseointegration of implants, excessive roughness can promote bacterial adhesion, affecting implants’ long-term stability [139]. Additionally, the use of nanoparticles in surface coatings can produce undesirable effects if not carefully assessed beforehand. For example, due to their small size and increased reactivity, they can influence the biological environment by interacting with immune cells and penetrating biological barriers. Most importantly, they can accumulate in organs, which can damage DNA, produce inflammatory reactions, or cause serious diseases. Moreover, even if methods are sought to remove nanoparticles from the human body, there is a risk that they could end up in the environment, affecting ecosystems and biodiversity. Thus, there is an urgent need for toxicological studies, so that the developed biodegradable materials can be used safely [140,141,142]. Despite these limitations, using nanotechnology for surface improvement remains one of the most promising approaches for next-generation spinal implants. With proper design and regulation, they can be safely implemented in clinical practice.

Recent advances are trending towards the adoption of nanostructured coatings for spinal implants, offering high osseointegration and antimicrobial properties. Meanwhile, 3D printing at the nanoscale represents an emerging field that will considerably enable the creation of patient-specific geometries with intricate, customizable, and porous structures. This alternative promotes bone growth and vascularization, leading to optimized bone fusion. Furthermore, by using appropriate materials (e.g., titanium, PEEK), the resulting 3D structures can exhibit greatly improved mechanical strength, yielding enhanced results [141].

**Figure 7:**
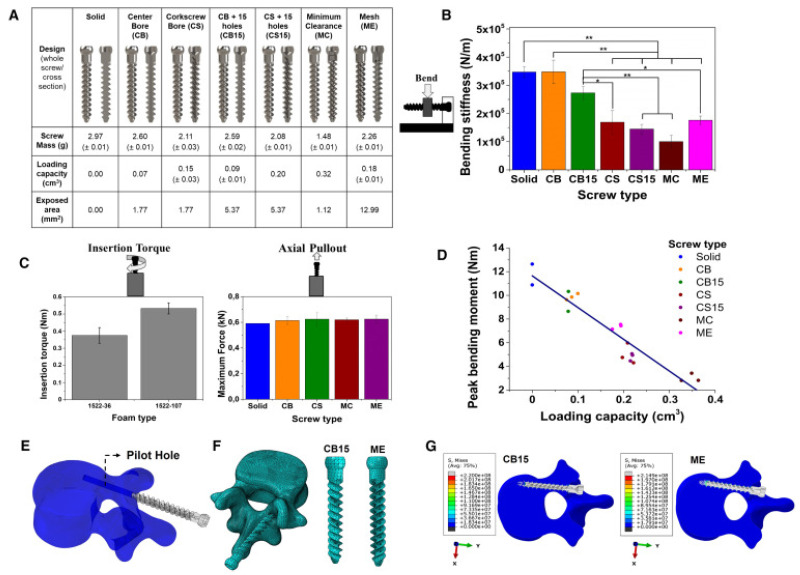
Three-dimensionally printed pedicle screw designed by Karavasili et al. (**A**) Seven 3D models of pedicle screws with varying internal geometries (solid, central channel, spiral, lattice) were tested, some with 15 rhomboidal perforations. (**B**) Bending stiffness was compared with statistically significant differences (* *p* < 0.05, ** *p* < 0.01). (**C**) Insertion torque and pull-out force were measured in two types of bone foam. (**D**) The correlation between the internal volume of the screw and the mechanical strength was analyzed. (**E**) The FEM model included an L2 vertebra with a pilot hole for screw insertion. (**F**) An adaptive mesh was generated for the analysis of CB15 and ME screws. (**G**) The von Mises stress distribution after insertion was evaluated. Reprinted from an open access source [144].

Thus, nanotechnology enables the production of nanocomposite materials, which, together with 3D printing, offer the possibility of obtaining innovative, versatile, multifunctional, and intelligent products. At the same time, the combination of these two technologies contributes to the development of spinal devices with improved surfaces and biodegradable materials, yielding significant clinical results [143,144]. However, materials selected for spinal devices should have high mechanical stiffness and prolonged biodegradation rates. Metals, ceramics, hard polymers, and composites are the most common biomaterials used in contemporary medical 3D printing technology, and they are stiff, making them ideal for orthopedic applications. This method offers enhanced printability, mechanical stability, and improved tissue integration for orthopedic fixation devices. Moreover, 3D-printed materials facilitate the fabrication of drug delivery systems and implants with excellent mechanical and biodegradable properties [145]. In a study conducted by Karavasili et al. [144], 3D-printed antibiotic-eluting pedicle screws (Figure 7) for antimicrobial prophylaxis in instrumented spinal fusion were developed. In this regard, a composite hydrogel was prepared in situ for antibiotic-sustained release (doxycycline) through the fenestrations of cannulated 3D-printed Ti-6Al-4V pedicle screws after placement within the vertebral body. Moreover, the hydrogel was enhanced using β-tricalcium phosphate (β-TCP) to improve screw osseointegration. In vitro tests demonstrated that 3D-printed implants exhibited a high antimicrobial effect against Staphylococcus aureus. Subsequently, they were successfully implanted in a porcine model, confirming the feasibility of their use in surgical applications. The researchers concluded that these screws could represent an innovative strategy for combating infections, while promoting implant osseointegration.

At the same time, developing spinal cages using 3D printing technology can represent an important aspect in improving spinal implants. This may allow for the customization of the implants as well as the creation of porosity on their surface, which promotes bone cell adhesion and the subsequent formation of new bone tissue. For example, 3D printing using materials like titanium can combine mechanical resilience and biological functionality, improving the body’s response to osseointegration [141]. Studies on 3D-printed titanium cages have shown a significantly lower early subsidence rate compared to PEEK cages. These results suggest that 3D-printed Ti cages are a viable and safe option [146].

Materials such as polylactide (PLA) and polycaprolactone (PCL) have been studied for the fabrication of spinal cages due to their gradual biodegradability, which allows for their replacement with newly formed bone tissue. This could considerably reduce the complications associated with permanent implants. Additionally, the formation of mixtures based on biodegradable polymers and materials with osteoconductive and osteoinductive properties (e.g., β-tricalcium phosphate, CaP, and the growth factor BMP-2) helps overcome the limitations on mechanical integrity, thereby improving spinal fusion rates [141].

Future perspectives in spinal implant designs aim to address some critical drawbacks, including infection control, poor osseointegration, and long-term biocompatibility issues. Three-dimensional printing technology is a promising strategy to create customizable implants tailored to patients’ needs, while the use of nanocomposite coatings can promote an increased antimicrobial effect and efficient osseointegration of implants. At the same time, biodegradable implants also represent an approach that reduces the occurrence of long-term complications. However, further studies are needed to optimize the balance between biocompatibility, mechanical stability, and bioactivity, which will support the transition to clinical trials.

## 5. Conclusions

Spinal implants are a necessity for restoring spinal stability and healing SD. However, materials such as metals, ceramics, and polymers still have drawbacks that need to be overcome. In this regard, nanotechnology represents an emerging strategy to enhance implant properties and develop new and innovative spinal implant types that promote osseointegration and SD healing.

Although there are recent advances and ongoing studies, extensive research is still needed to optimize the materials used to manufacture spinal implants. Thus, extensive and rigorous clinical and preclinical studies are needed to better understand how the materials interact with the physiological environment, minimize post-implantation complications, and observe the long-term integration of implants with bone tissue. At the same time, decreasing the risk of infection in implants would increase implant success rates. Therefore, researchers should focus on developing innovative coatings with antimicrobial effects that help overcome this drawback. Future studies should explore the use of nanotechnology as well as 3D printing fabrication, which offers significant opportunities for implant customization. Additionally, the use of bioresorbable implants could lead to a breakthrough in spinal surgery, as it may facilitate proper healing without the need for revision procedures.

## Figures and Tables

**Table 1:** Spine physiological loadings type.

Spinal Region	Loading Type	Activity	Typical Magnitude	Observation	Refs.
Cervical	Compression	Flexion	550 N	Maximum for C4–C5 and C7–T1	[34]
	Bending Moments	Flexion Extension	1.6–3.5 (Nm)	These estimation times were evaluated in vitro	[35]
	Shear	Flexion	100 N	These forces increase in flexion for C0–C3 and decrease at C6–C7 and C7–T1	
Thoracic	Compression	Relaxed	283 (N)	-	[36]
		Flexion	752 (N)		
		Lateral Bending	438 (N)		
Lumbar	Compression	Standing	596 (N)	Axial load during regular standing	[37]
		Bending Forward (30°)	1271 (N)	It was observed that compression increases with forward bending	
		Bending Forward (90°)	2195 (N)	A peak load was observed during the bending	
		Walking	966 (N)	Moderate load	
		Climbing Stairs	1206 (N)	Increased load	
		Getting up	2384 (N)	Highest load	
	Bending Moments	Flexion Extension	2.6–10 (Nm)	This estimation was evaluated in vitro	[35,36]

**Table 4:** Overview of the coating effects on spinal implants. Created based on the information from [136].

Implant Type	Implantable Material	Surface Modification Technique	Observations
Interbody	Titanium	Surface roughening	Improve initial fixation Stimulates osteoblast differentiation Leads to better bone formation
		Porous surface	Reduce stress shielding Promote bone ingrowth Increased porosity leads to wear debris
		Chemical Modification	Enhances osseointegration Mimic bone’s chemical composition
	PEEK	Coating with composite materials	Improve osseointegration Enhances bone growth
		Porous surface	Mimic the structure of the trabecular bone Improves cell attachment and bone ingrowth
Pedicle Screws	Titanium	Roughened Titanium	Increase pull-out strength Promote osteoblast activity Reduce the risk of loosening
	Titanium Stainless steel	Hydroxyapatite coating	Enhance bone deposition along the screw surface Improve osseointegration Reduce loosening rates
	Titanium alloy	Carbon Fiber-Reinforced PEEK (CF/PEEK)	Reduce imaging artifacts Improve the postoperative assessment They are costly and not widely adopted
	Titanium	Gold nanoparticles	Enhance osseointegration Promote osteogenic differentiation
		Silver nanoparticles	Provide antibacterial properties Reduce the risk of infections
